# From Fear to Function: How Physiotherapists Navigate Psychological Challenges in Patients Undergoing Rehabilitation After ACL Reconstruction

**DOI:** 10.1177/19417381261471381

**Published:** 2026-09-24

**Authors:** Ingunn Fleten Mo, Anne Gro Heyn Faleide, Trine Hysing-Dahl, Liv Heide Magnussen, Per-Henrik Randsborg, Rob Bogie, Eivind Inderhaug, Nicky van Melick

**Affiliations:** Haraldsplass Deaconess Hospital, Bergen, Norway, University of Bergen, Bergen, Norway, Sports Traumatology and Arthroscopy Research group, Bergen, Norway; Haraldsplass Deaconess Hospital, Bergen, Norway, University of Bergen, Bergen, Norway, Sports Traumatology and Arthroscopy Research group, Bergen, Norway; Western Norway University of Applied Sciences, Bergen, Norway, Sports Traumatology and Arthroscopy Research group, Bergen, Norway; Western Norway University of Applied Sciences, Bergen, Norway; Akershus University Hospital, Lørenskog, Norway, University of Oslo, Oslo, Norway; Anna TopSupport, Geldrop, North Brabant, the Netherlands; University of Bergen, Bergen, Norway, Sports Traumatology and Arthroscopy Research group, Bergen, Norway, Haukeland University Hospital, Bergen, Norway; Anna TopSupport, Geldrop, North Brabant, the Netherlands

**Keywords:** ACL rehabilitation, physiotherapy, psychological factors, psychological readiness, return to sport

## Abstract

**Background:**

Psychological readiness plays a critical role in return to sport (RTS) outcomes after anterior cruciate ligament reconstruction (ACLR), yet physiotherapists (PTs) often feel unprepared to support this aspect of recovery. Although clinical guidelines advocate for psychological strategies, practical tools and structured interventions remain scarce.

**Hypothesis:**

Experienced PTs will actively use psychological strategies during rehabilitation to support patient readiness for RTS. PTs perceive themselves as playing a central role in addressing psychological barriers.

**Study Design:**

Qualitative study using semi-structured focus group interviews.

**Level of Evidence:**

Level 5.

**Methods:**

A total of 21 experienced PTs were recruited based on their involvement in ACLR rehabilitation. Three focus group interviews were conducted in each country using a semi-structured guide, developed in English and translated into Norwegian/Dutch. Interviews conducted via video call were recorded, transcribed verbatim, and analyzed using systematic text condensation to identify key themes.

**Results:**

PTs reported investing considerable time in building therapeutic alliances, enabling them to support psychological readiness throughout ACLR rehabilitation. Common strategies included individualized communication, goal-setting, reassurance through milestone tracking, and graded exposure to sport-specific movements. PTs also adapted their approaches based on patient responses. However, barriers such as time constraints, lack of formal psychological training, and organizational disruptions were identified.

**Conclusion:**

PTs play an essential role in addressing psychological readiness in ACLR rehabilitation. Key strategies include building alliances, individualized support, and gradually reintroducing patients to sport-specific activities. Despite their efforts, limited psychological training and structural challenges can hinder optimal care. Addressing these gaps may enhance rehabilitation outcomes and RTS success.

**Clinical Relevance:**

This study provides practical insight into how PTs manage psychological challenges in ACLR rehabilitation. Findings highlight the need for more formal psychological training and integrated interdisciplinary approaches to better support recovery and improve RTS outcomes.

Rehabilitation after anterior cruciate ligament (ACL) reconstruction (ACLR) has focused traditionally on restoring physical function, emphasizing strength, mobility, and sport-specific movements. However, recent years have seen a growing recognition of the psychological factors associated with ACL injuries, including fear of reinjury, anxiety, frustration, loss of athletic identity, and the importance of psychological readiness to return to sport(RTS).^7,8,21,32,38,39^ The latter is defined as an athlete’s mental preparedness to resume sport-specific activities, encompassing their perception of cognitive appraisal, emotional responses, and behavioral readiness.^34^ Psychological factors can impact rehabilitation significantly, influencing RTS rates, risk of reinjury, and overall recovery outcomes.^3,11,23,27,29,30^

As awareness of these psychological factors increases in the field of sports medicine,^35^ the role of physiotherapists (PTs) is evolving beyond traditional rehabilitation to encompass a more holistic, biopsychosocial approach to care. Within this framework, building a strong therapeutic alliance - the collaborative and trusting relationship between patient and physiotherapist, built on shared goals, tasks, and emotional bond - is essential to support engagement and positive rehabilitation outcomes.^5,15^ Current clinical guidelines emphasize the importance of integrating psychological factors into ACL rehabilitation.^12,37^ Strategies to improve psychological outcomes include goal setting, gradual exposure, arousal regulation, information, and visualization.^2,9,10,14,21,22,25^ However, many PTs report feeling underprepared to address these challenges effectively.^9,31^ This may be due to the lack of high-quality evidence supporting effective psychological interventions,^18,36^ leaving PTs to rely on experience and informal strategies rather than adopting structured, evidence-based approaches.

Given the growing recognition of the importance of psychological factors in rehabilitation and the lack of structured interventions to address them, it is essential to understand how experienced PTs navigate the psychological challenges faced by patients during in ACLR rehabilitation. By improving the understanding of how to help patients overcome psychological barriers, PTs can better personalize rehabilitation, contribute to safer RTS decisions and improve long-term knee health. Ultimately, these insights may inform more comprehensive rehabilitation protocols that address both the psychological and physical aspects of recovery.

The aim of this study was to explore how PTs address psychological challenges after ACLR, investigate how they perceive their role in enhancing psychological readiness, and identify the obstacles they may encounter. Furthermore, as this qualitative study was conducted in both Norway and the Netherlands, we aim to capture a broad range of perspectives and insights across nations and diverse healthcare settings.

## Methods

The project is a collaboration between Haraldsplass Deaconess Hospital (HDS, Norway), Sports Traumatology and Arthroscopy Research group (STAR, Norway) and Anna TopSupport (the Netherlands).

## Participants and Recruitment

The PTs were selected strategically through the research groups’ networks in their respective countries. Norwegian PTs were contacted by the first author, while Dutch PTs were contacted by the last author. The PTs were informed about the study and invited to participate. A total of 33 PTs (14 Dutch) were contacted, of whom 7 (3 Dutch) declined to participate due to a busy schedule, and 5 (0 Dutch) did not reply to our requests. Eventually, a sample of 21 PTs (11 Dutch) was included. To ensure a rich data variation, PTs who had regularly treated patients after ACLR the last 5 years (approximately >10 patients per year) were included. The participating PTs reported treating patients across a wide range of ages, activity levels, and sports. Demographic data are presented in Table 1. All PTs worked independently of the clinics conducting the study. Three focus groups were conducted in each country, with a comparable number of PTs, similar participant characteristics, and a diverse geographical distribution in each interview.

**Table 1.** Demographics of participants (N = 21)

Characteristics	The Netherlands (n = 11)	Norway (n = 10)
Age, years	40 (32-53)	43 (35-55)
Female sex, n (%)	6 (54.5)	4 (40)
Experience, years	17 (8-25)	19 (9-25)
ACL patients per year, n	45 (8-120)	25 (10-50)
Education*^a^*		
Bachelor’s degree in physiotherapy, n (%)	0 (0)	4 (40)
Master’s degree in sports physiotherapy or specialist in sports physiotherapy, n (%)	10 (91)	3 (30)
Master’s degree in manual therapy, n (%)	1 (9)	3 (30)

Results given as median (range), unless otherwise stated. ACL, anterior cruciate ligament; DPT, Doctor of Physical Therapy; PT, physiotherapist.

a While none of the participating PTs hold a DPT degree, the educational level and clinical scope of practice in Norway and the Netherlands are assumed to be comparable with that of DPT-trained clinicians in the United States.

## Data Collection

A semi-structured interview guide was developed, inviting the PTs to express their experiences regarding psychological factors in ACLR rehabilitation. A Norwegian sports psychologist was consulted when forming the interview guide, to ensure a comprehensive understanding of terminology used. The interview guide was developed in English, before being translated into Norwegian and Dutch by PTs fluent in all 3 languages. The overarching themes were (1) how psychological responses may impact rehabilitation and RTS for ACL patients, (2) which strategies are found helpful or inhibiting in achieving postoperative goals after ACLR, and (3) to what extent PTs are aware of psychological responses and their potential role in addressing psychological barriers during ACLR rehabilitation.

Data collection occurred between March and October 2024. The focus group interviews were conducted by the authors (healthcare professionals) in Norway and in the Netherlands, via video link, and recorded by the first and last author. Between 1 and 3 members of the research group were present at each interview. I.F.M. is a PhD candidate and PT in the orthopaedic field. L.H.M. is a professor of physiotherapy with extensive knowledge in qualitative research. A.G.H.F. is a postdoctoral researcher and PT, and N.v.M. is a postdoctoral researcher and former sport PT with 15 years of experience in ACLR rehabilitation. E.I. and R.B. are orthopaedic surgeons with >11 years of experience in ACLR surgery. All members of the research group have extensive knowledge of ACL research. There were no direct connections between the informants and the authors of the study.

All interviews were recorded and transcribed verbatim. Short field notes were made after each interview to validate transcript content in further analyses.

## Data Analyses

The data were analyzed using systematic text condensation (STC) - a rigorous, stepwise method for thematic analysis designed to identify and synthesize key experiences and perspectives across multiple participants^26^; STC has been used in other studies exploring musculoskeletal injuries.^16,17,20,28^ STC is particularly suitable for qualitative studies in clinical settings because it allows researchers to systematically condense rich, detailed accounts into clear, overarching themes that capture the essence of participants’ experiences, making the findings accessible and meaningful to practitioners.^26^ STC involves 4 main steps. (1) Getting an overall impression: the entire dataset was reviewed thoroughly to identify preliminary themes and patterns. (2) Identifying and categorizing meaning units: statements and excerpts related to PTs’ experiences with psychological readiness after ACLR were highlighted and organized into code groups to structure the material systematically. (3) Condensing the content: each code group was summarized into concise, meaningful condensates, emphasizing the most essential aspects of the material. (4) Synthesizing the findings: the condensed summaries were integrated to develop clear, comprehensive descriptions of each theme and subgroup, providing an overarching understanding of the PTs’ perspectives.^26^ Preliminary analyses were performed after 3 focus group interviews to allow for adjustments of the interview guide;^26^ no changes were necessary. Steps 1 to 3 were performed separately in Norway and the Netherlands. The fourth step was done in unison between the first and last author, discussing until a common agreement was reached, forming result categories from our separate condensates. Preconceptions were discussed continuously in the project group throughout the study process. This approach ensures that the analysis remains systematic and transparent, allowing sports and rehabilitation professionals to understand how psychological readiness is addressed by PTs in ACLR rehabilitation.

## Results

The 6 focus group interviews averaged 85 minutes, yielding 142 pages of transcribed text. Data analysis identified 4 result categories.

### Theme 1 - Building Trust and Emotional Resilience Through Therapeutic Alliance

PTs described a strong therapeutic alliance as fundamental to successful ACL rehabilitation. The stronger the alliance, the easier it became to motivate patients through the lengthy process. Many PTs reported investing significant time in building trust, allowing them to gradually introduce more challenging tasks. Most PTs viewed their years of experience as an important factor in guiding patients after ACL surgery, allowing them to better recognize patterns and anticipate patient needs. Gaining patients’ trust in their competence was also viewed as essential for fostering psychological readiness, making the patients feel more secure and confident. A male Norwegian PT stated:

“*When I think of myself today versus 10 years ago, there is a major difference because you need to get that experience and become confident in that role of facing the challenges of rehab, because you see different sorts of “normal” rehabilitation processes and that’s not something you can necessarily just buy from a course or read about. So, you have to gradually become more comfortable in that role - through experience.*”

PTs also highlighted the importance of addressing emotions early, normalizing frustration and sadness, and validating patients’ experiences. Some PTs even scheduled extra appointments specifically for this purpose, highlighting the importance of the therapeutic alliance in fostering emotional resilience. A female Norwegian PT said:

“*I think it’s important to allow them to be frustrated, and to be sad about the situation. And I tell them that it’s completely normal to feel that way, and that it’s OK. And then we have to try to talk our way through it.*”

### Theme 2 - Strategies for Improving Psychological Readiness and Motivation

PTs described using *graded exposure* to gradually reintroduce patients to activities and environments associated with their injury, thereby rebuilding confidence. Some conducted exercises at the arena where the injury occurred, used video clips of the incident, or encouraged visualization to address fear. They also emphasized the psychological impact of *physical progression*, using regular knee function testing and small, structured goals to maintain motivation. Contextualizing these goals helped patients understand how each exercise contributed to both physical and mental recovery. A male Norwegian PT noted:

“*Having these physical goals helps them experience a sense of achievement - almost forcing a feeling of mastery on them. Small milestones are really just little drops of success they can experience along the way, and together, they might start building a stronger belief in their own abilities.*”

PTs also described how information and structure create safety in what can feel like a chaotic process. They achieve this by providing information throughout the injury process, normalizing symptoms, answering questions and offering reality-oriented guidance. Well-informed patients were generally less anxious and more motivated.

### Theme 3 - Managing Internal and External Influences

PTs observed that motivation and confidence fluctuate throughout ACL rehabilitation, influenced by pain, setbacks, and personal coping styles. Some patients displayed a “victor” mindset, while others struggled with motivation and setbacks, taking on a “victim*”* mindset instead. PTs emphasized the need to adapt continuously to these emotional shifts. A female Dutch PT explained:

“*I think there are very few ACL rehabilitation patients who don’t hit a wall at some point - whether it’s in motivation, progress, time, or the constant need to structure everything around their recovery and rehabilitation. I do believe it’s highly patient-dependent.*”

Group training was described as both motivating and supportive, helping patients share experiences and find encouragement from peers. However, PTs noted that social comparison could have a negative impact if patients compared themselves with higher-performing peers.

External influences - such as parents, coaches, and teammates - were also mentioned frequently. Although often well-intentioned, they could increase emotional pressure or hinder progress. PTs emphasized the importance of setting clear boundaries early in the rehabilitation process. A male Dutch PT pointed out:

“*There are always people who think they have influence over the recovery process - the neighbor, a teammate, the coach, or even the technical director. Clubs often want the process to move faster than what the medical side considers wise or safe, at least in my experience. I think making clear agreements upfront really helps manage this pressure effectively.*”

### Theme 4 - Physiotherapists’ Role in Improving Psychological Readiness

Most PTs viewed their primary role as supporting physical recovery but acknowledged their parallel role in enhancing psychological readiness. Many felt confident in addressing mental factors, drawing on experience, sports-specific education, or cognitive therapy courses. Others expressed a wish for more knowledge on applying these approaches effectively. Their close and consistent contact with patients was seen as a unique opportunity to support psychological readiness throughout rehabilitation.

PTs reported using patient-reported outcome measures (PROMs) such as the ACL-Return to Sport after Injury (ACL-RSI) and the Photograph Series of Sports Activities for ACL Reconstruction (PHOSA-ACLR) to assess mental factors, although practices varied. Some applied these tools consistently; others relied mainly on clinical judgment and open dialogue, finding direct conversations often more insightful than standardized tools.

Collaboration with sports psychologists was considered beneficial but often limited by availability and insurance coverage. Long waiting lists and lack of access were described as barriers to optimal care. A Norwegian male PT expressed:

“*In primary healthcare we don’t have those resources [that professional athletes do]. If I needed a sport psychologist or psychologist for my patient, then. . .we don’t have access to that. It’s not a thing here [where my clinic is at], and in primary healthcare you can just forget about it, right? The waiting lists are endless!*”

## Discussion

This study explored how Norwegian and Dutch PTs experienced in ACLR rehabilitation perceive their role in enhancing psychological readiness of patients with ACLRs and gives rich insight to different strategies used by PTs for improving psychological readiness in ACLR rehabilitation.

### What Does This Study Add?

The important role of PTs in enhancing psychological readiness was evident in this study. For some, it was an explicit focus, while others described doing so implicitly through physical rehabilitation. To a large extent, they followed current recommendations for enhancing psychological readiness, such as goal setting, graded exposure, information, and visualization.^2,9,14,21,22,25^ This aligns with research showing that structured mental skills training can facilitate recovery and improve mental health and performance.^4,6,13,24^ Some PTs also used recontextualization as part of exposure - conducting exercises in settings where the injury occurred - or used video footage of the injury as a therapeutic tool.

Despite the growing focus on psychological readiness, many PTs still lack formal training in psychological intervention.^9,19,31^ Most PTs in the current study feel confident in improving psychological readiness by using their clinical toolbox, but, consistent with findings from a recent survey by Cederström et al,^9^ some wanted more structured knowledge and training in managing psychological barriers. This is unsurprising, as a recent meta-analysis found limited high-quality evidence supporting specific psychological interventions after ACLR.^18^ Consequently, PTs often rely on clinical experience rather than standardized, evidence-based approaches, underlining the need for stronger education and clearer research-based guidance.

All PTs in this study treat a high volume of ACL injuries annually and were recruited strategically by the authors from our networks in Norway and the Netherlands. Many developed their expertise through years of experience, sports-specific education, and courses on psychological readiness. However, not all PTs treating ACL injuries see such volumes, which may limit their experience and confidence. This highlights the importance of continuing education and literature engagement to ensure evidence-based practice. Yet, Piussi et al^31^ found that PTs rarely mentioned reading academic papers or attending scientific courses - an observation echoed in our findings, where some expressed uncertainty about where to find reliable information on enhancing psychological readiness. This points to an ongoing gap between research and clinical practice.

Goal setting is a well-established strategy for promoting motivation and psychological readiness^2,12,33^ and is used commonly in clinical practice to keep patients motivated throughout rehabilitation and improving psychological issues.^1,9,31^ Our findings show that PTs deliberately set small, progressive physical goals to foster mastery and confidence. Importantly, these goals were contextualized individually, helping patients link early-stage tasks (e.g., regaining full knee extension) to sport-specific performance (e.g., kicking a ball). This strengthens both compliance and trust in the rehabilitation process. Hence, deliberate goal setting not only supports psychological readiness but also reinforces patients’ trust in physical performance.

Current ACLR guidelines recommend assessing psychological readiness before RTS.^12,37^ The PTs reported using PROMs such as the (short) ACL-RSI and PHOSA-ACLR for this purpose, especially in RTS evaluations. However, many emphasized that clinical judgment remains their most important tool, and several requested clearer guidance on administration and interpretation of PROMs. Establishing standardized protocols for integrating psychological assessments into rehabilitation could enhance decision-making and RTS safety.

PTs emphasized that, while few ACL patients required referral to mental health specialists, collaboration with (sports) psychologists was valuable when needed. However, long waiting times, limited availability, and lack of insurance coverage were identified as key barriers. This challenge underscores the need for PTs to actively monitor and address psychological factors themselves and to develop skills for early detection and management to prevent escalation of mental barriers.

### Methodological Considerations

This study followed a transparent and reproducible design based on Malterud’s STC.^26^ We interviewed experienced PTs in ACLR rehabilitation across 2 countries, providing diverse perspectives on psychological factors and the role of PTs. All participants worked independently of the study clinics, reducing institutional bias.

Conducting the study in both Norway and the Netherlands enriched the data through varied healthcare perspectives. While differences in system structure and culture could have influenced findings, this was not evident in the analysis. Although focus groups were smaller than Malterud’s recommended 5 to 8 participants (average 3.5 per group),^26^ this allowed for more in-depth discussions, and conducting 6 groups provided sufficient information power. While ideally each STC step should be conducted sequentially with consensus reached before proceeding,^26^ our approach prioritized consistency and reflexivity. Preconceptions and interpretations were discussed continuously among the research team to strengthen credibility.

The project group included clinicians and researchers with extensive ACL expertise, which was a strength in developing a relevant interview guide. However, this may also have introduced bias, potentially shaping the questions or analysis. Some interviewers were employed at hospitals referring patients to PTs in primary care, but most participants worked outside these regions, minimizing influence.

Finally, this study explores PTs’ perceptions only. There may be a gap between how PTs believe they provide psychological support and how athletes perceive receiving it. Even if a PT feels confident and supportive, the athlete may not experience it as such - a crucial discrepancy that future studies should explore from the athlete’s perspective.

## Conclusion

This study identifies the critical role of PTs in addressing the psychological challenges of ACLR rehabilitation. Through graded exposure, goal setting, patient education, and strong therapeutic alliances, PTs support patients in building confidence, motivation, and psychological readiness for RTS. However, managing emotional fluctuations, external pressure, and psychological readiness assessments remains challenging, with varied approaches to mental health support. These findings suggest that sports medicine and healthcare professionals should place greater emphasis on integrating psychological strategies into rehabilitation. While PTs acknowledge their role in fostering psychological readiness, limited access to specialized mental health resources and a need for further training persist. Future research should explore structured psychological interventions and their impact on rehabilitation outcomes to enhance patient support beyond physical recovery.
